# The right coronary artery in the heart of chinchilla (*Chinchilla laniger* Molina)

**DOI:** 10.1007/s11259-022-10035-4

**Published:** 2022-11-16

**Authors:** Jacek Kuchinka, Małgorzata Radzimirska, Dariusz Banaś, Elżbieta Nowak, Aleksander Szczurkowski

**Affiliations:** 1 Department of Medical Biology, Institute of Biology, Jan Kochanowski University in Kielce, 7 Uniwersytecka St., 25-406 Kielce, Poland; 2 Department of Atomic Physics and Nanophysics, Jan Kochanowski University, Kielce 7 Uniwersytecka St., 25-406 Kielce, Poland; 3 Department of Pathomorphology, Histology and Forensic Medicine, Collegium Medicum, Jan Kochanowski University in Kielce, 19a IX Wieków Kielc St, 25-516 Kielce, Poland

**Keywords:** Anatomy, Coronary arterial system, Chinchilla

## Abstract

The pattern of normal coronary vascularization in a mammalian heart includes the presence of both right and left coronary arteries. According to the literature data, the presence of single major coronary arteries is mainly related to cardiac abnormalities. Previously it has been reported that the right coronary artery is absent in the coronary vascularization of the heart in the chinchilla. Our research was carried out on thirty chinchillas (*Chinchilla laniger* Molina). The coronary vessels were filled with colored latex to render them visible. The examinations were supplemented additionally with the use of microcomputed tomography with arterial contrast. Our study demonstrates its undoubtedly presence of the right coronary artery. In all subjects the right coronary artery was present, as was the left coronary artery. Two types of right coronary artery were found. Our results indicate that the normal pattern of coronary vascularization of heart in chinchilla includes both the right and left coronary arteries. An open question remains the presence of single coronary artery is a normal pattern of cardiac arterial vascularization in chinchilla.

## Introduction

The coronary circulation is responsible for the blood flow in the vessels that supply miocardiocytes with oxygen and nutrient-rich blood, while removing carbon dioxide and metabolic byproducts. In mammals, right and left aortic sinuses give rise to the main coronary arteries (right and left, respectively). This anatomy is considered normal and is associated with the presence of the right and left coronary ostia (Fernandez et al. 2007; Hill and Jaizzo 2009). The arrangement of the coronary vessels in chinchilla follows the same pattern.

The coronary circulation has been the subject of numerous studies, in humans (Friedman et al. 2007; Noestelthaller et al. 2007; Singh et al. 2017; Yan et al. 2018) and in numerous species of mammals, including rabbit (Day and Johnson 1958; Podesser et al. 1997; Aksoy and Karadag 2002), beaver (Bisaillon 1981), monkey (Buss et al. 1982), domestic animals (Nickel et al. 1981), donkey (Dursun 1977; Ozgel et al. 2004), cat (Vladova 2005), and mouse (Yoldas et al. 2010). A precise research methodology for visualizing the morphology of these coronary vessels in rats has been developed using corrosion preparations (Ślusarczyk et al. 2004) and Kainuma et al. (2017) have given a detailed description of these vessels in this species.

Variation in the arrangement of the coronary arteries has been widely reported in human (Koizumi et al. 2000; Yan et al. 2018) and in numerous animal species: cattle (Cerny 1976), Syrian hamster (Durán et al. 2006, 2007a, b), dog (Noestelthaller et al. 2007), mouse (Lopez-Garcia et al. 2016), dog, hamster, cow, horse, and pig (Scansen 2017).

In recent years, the chinchilla has become an important experimental species and clinical reports refer to cardiac murmurs and ventricular septal defects (Heatley 2009; Linde et al. 2004; Pignon et al. 2012). The coronary vascularization in this species has been described (Özdemir et al. 2008). Many authors have referred to the presented above research: Heatley (2009), Harkness et al. (2010), Yoldas et al. (2010), Martinez-Pereira and Rickes (2011), Suckow et al. (2012), Correia-Oliveira et al. (2014), Barszcz et al. (2013, 2014, 2017, 2019), Kupczyńska et al. (2015), Scansen (2017), Martonos et al. (2017, 2018, 2019), Magariños et al. (2018), Turner et al. (2018).

The results of the study “The right coronary artery is absent in the chinchilla (Chinchilla lanigera)” by Özdemir et al. (2008) strongly suggest that the right coronary artery is always absent in this species. As this finding was completely inconsistent with our observations, we reexamined the presence of the right coronary artery in the hearts of healthy adult chinchillas. Our research is related to the normal course of the coronary vessels in chinchilla and does not concern any developmental anomalies or inbreeding animals.

## Materials and methods

The research was carried out on 29 adult individuals at approximately nine months of age and on one dead newborn chinchilla (*Chinchilla laniger* Molina). Both the age and sex of the animals were determined by professional breeders and confirmed by us in autopsy. The fetal sex was determined to be female at the perinatal age. The animals were of both sexes (thirteen males and seventeen females) and weighed 430 g to 550 g. They were obtained from a professional reproductive farm E-012 address: Skoki 69A Poland and were killed for fur by qualified personnel according to the Polish law. Immediately after slaughter, the thoracic and abdominal cavities were opened, and the abdominal aorta was cannulated. The wall of the right atrium of the heart was then cut. Opening the right atrium (right auricle) of the heart helps the flow of the solution through the circulatory system.

While controlling the pressure with a semiautomatic syringe, the artery system was flushed with cold 0.9% saline solution mixed with 5000 IU of heparin (Heparinum, Polfa, Warsaw). It was then carefully filled with acrylic latex (LBS 3060 Synthos-Manors, Poland) colored with red pigment (Pigment-Mix-Inchem, Poland). The level of latex filling of the arterial coronary vessels was controlled by observing filled smallest coronary vessels on the surface of the ventricles and atria (about 2 min.). The material was fixed in 7% formaldehyde solution for about a week. After polymerization, the hearts were examined under a stereoscopic microscope (SMZ 800 Nikon Japan). The corrosion specimen (n = 3, including partial corrosion = 2) was prepared using the Duracryl® Plus Spofa – Dental a.s. Czech Republic. Photographic documentation was performed using a Nikon Digital Sight DS-L3 system. Measurements and calculations were carried out using NIS Elements imaging software (version: 4.11.00). A micro-CT examination (n = 3) was performed using Skyscan 1172 micro-CT scanner, 20 – 100 kV X-ray tube. The X-ray contrast agent consisted of 45% barium sulfate (Medana Polpharma, Poland) and 55% latex (Sedlmayr and Wittmer 2002). The results were described using veterinary anatomical nomenclature (NAV 2017). The research conformed with the requirements of the Polish Act for the Protection of Animals Used for Scientific or Educational Purposes (15 January 2015). Studies of tissues obtained postmortem do not require the approval of an ethics committee.

## Results

**Fig. 1:**
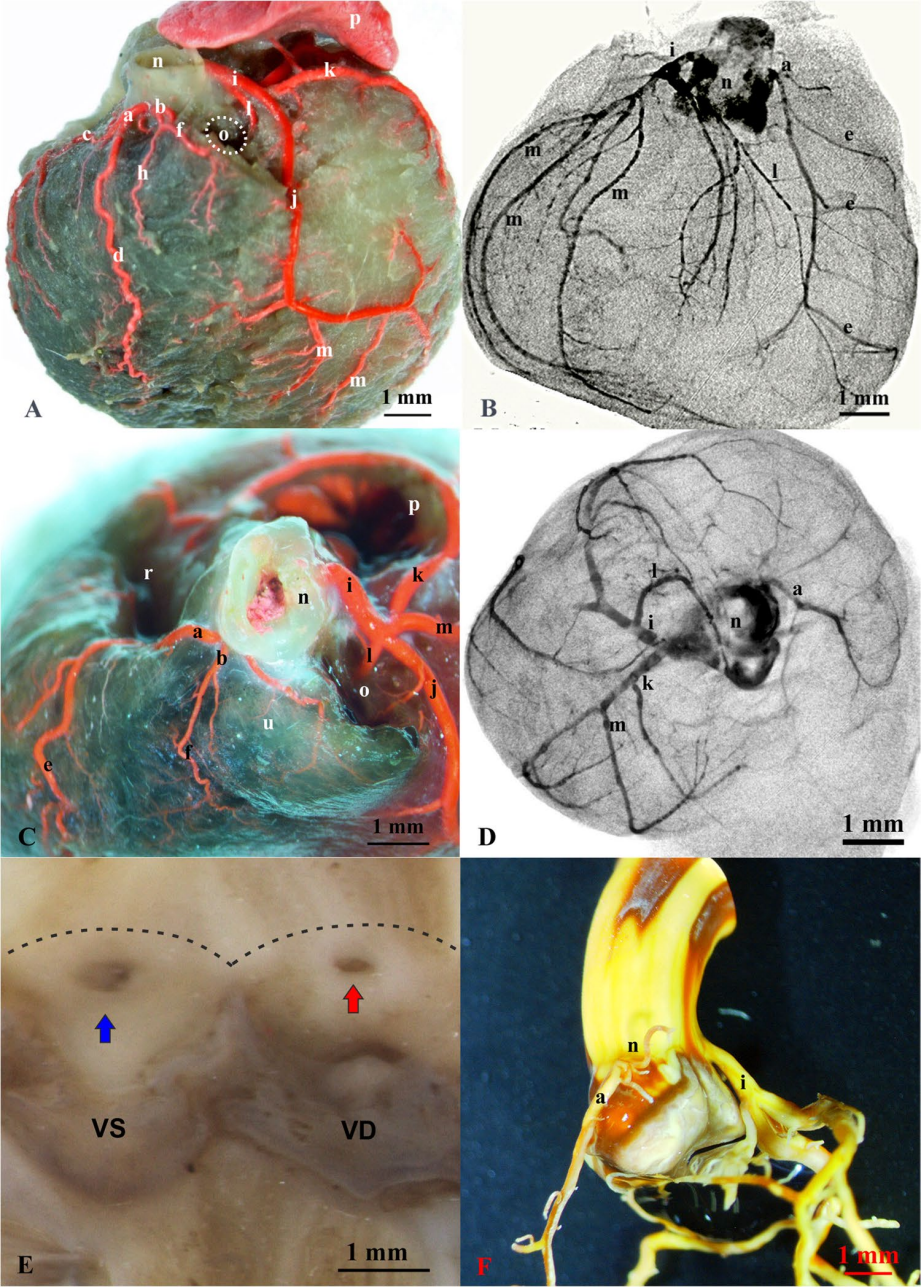
Arterial vascularization of the chinchilla’s heart. **A**—gradual corrosion arterial specimens (right antero-lateral aspect), **C** – gradual corrosion arterial specimens (basal aspect); **B**, **D** – micro-CT view of coronary arteries (MIP inverse – maximum intensity projection); **E** – internal openings of the coronary arteries (RCA and LCA) – macroscopic view, dashed line – sinotubular junction; **F** – aortal bulb with RCA and LCA corrosion cast

**Fig. 2:**
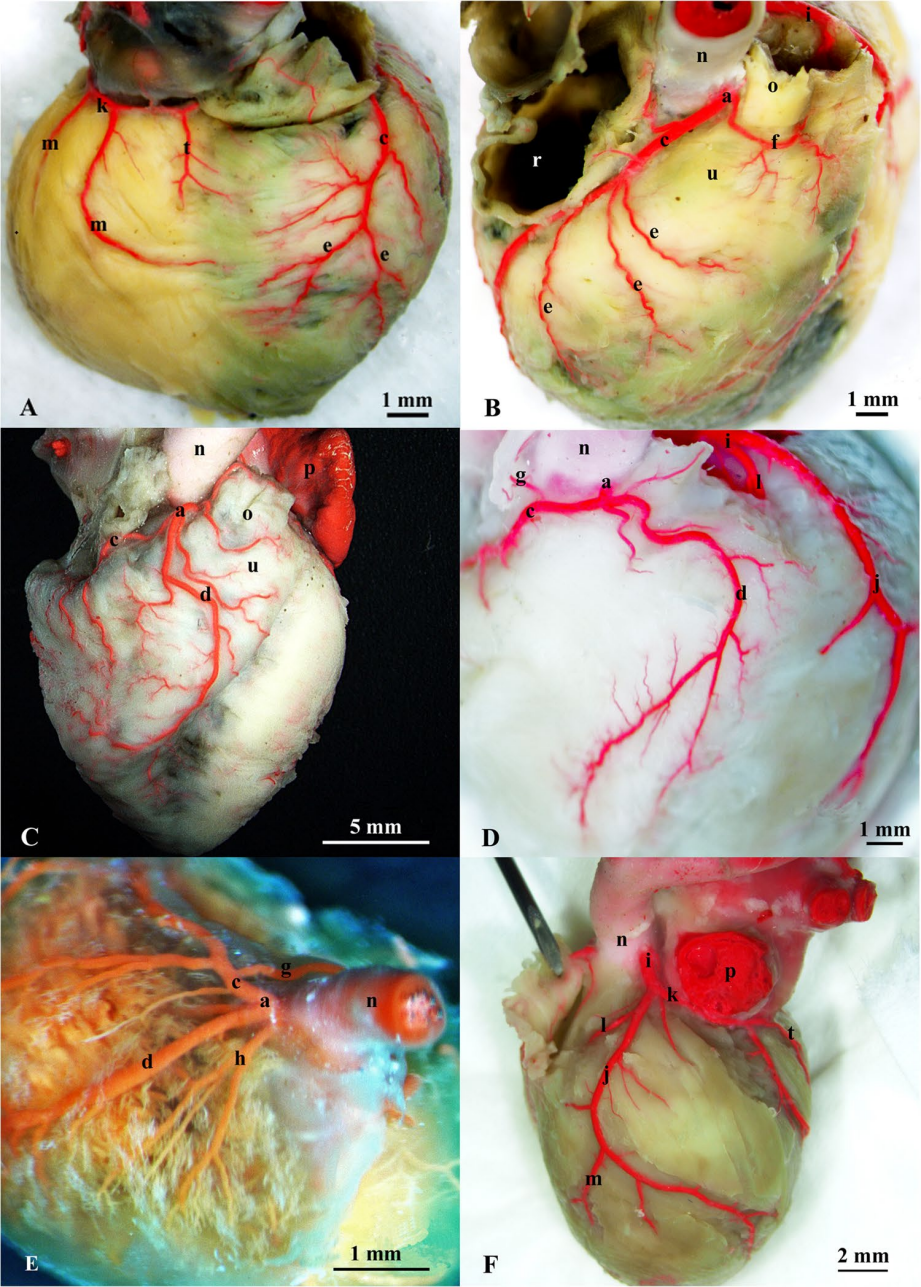
Arterial vascularization of the chinchilla’s heart. **A** – branches of the coronary arteries (atrial surface); **B** – right coronary artery (RCA) – type I (right antero-lateral aspect); **C**—right coronary artery (RCA) – type II (right antero-lateral aspect); **D**—right coronary artery (RCA) – intermediate variant (right antero-lateral aspect); **E**—right coronary artery (RCA) – fetal gradual corrosion specimen; **F** – left coronary artery (LCA) – vascular specimen (left lateral view). a – right coronary artery (RCA). b – accessory right coronary artery. c—right circumflex branch of the RCA. d – marginal branch of the right ventricle. e – ventricular branches of the RCA. f – paraconal branch of the pulmonary trunk. g – sinoatrial nodal branch. h – arterial branches. i – left coronary artery (LCA). j – interventricular paraconal branch of the LCA. k – left circumflex branch of the LCA. l – septal branch. l*—distal part of the septal branch. m – interventricular branches of the LCA. n – aortal bulb. o – pulmonary trunk, place of the cut-off is indicated by a dashed line. p – left auricle of atrium. r – right auricle of atrium (cut off). s – atrioventricular nodal branch. t – subsinuosal interventricular branch. u – conus of pulmonary trunk. VD – right semilunar cusp. VS – sinister semilunar cusp

Analysis of the coronary vascular system of the chinchilla heart showed in all cases the unambiguous presence of both left coronary artery—LCA (*a. coronaria sinistra*) and right coronary artery – RCA (*a. coronaria dextra*) (Figs. 1, 2).

The right coronary artery trunk ran subepicardial. The main vessels branching from the trunk were undulating. They plunged in sections deep into the heart muscle, and the subsequent side branches undulated, though definitely within the superficial layer of the muscle. Further vessels were clearly directed deeper into the muscle, giving way to brush-like branched end vessels. Observations of the left coronary artery (*a. coronaria sinistra*) with its branches showed a similar course. In chinchillas, epicardial fat surrounding coronary arteries is usually not observed.

The right coronary artery (*a. coronaria dextra*) showed high morphological variability in the trunk, particularly in its length, its branches, and course, as well as in terms of the vascularized area (Figs. 1A-D, 2A-D). The diameter of the RCA was 0.38 mm (± 0.08). In all the examined individuals, the RCA began with an orifice (*ostium*) in the right sinus (*sinus dexter*) of the aortic valve on the level of its sino-tubular junction (Fig. 1E). In a few cases (n = 4), the opening on the level of the aortic bulb (*bulbus aortae*) wall was divided, giving rise to smaller additional branches of the RCA (Fig. 1A, C). In all the cases (n = 30), the RCA split by giving off one to three proximal branches and a similar number of distal branches of the right atrium (*atrium dexter*) (Figs. 1A-F, 2B-D). A variable number of branches was observed around the pulmonary trunk (*truncus pulmonaris*) arterial cone (*conus arteriosus*). In a few cases, these vessels, as small accessory coronary arteries, supplied the arterial cone or the pulmonary trunk, departing separately from the aortic bulb (Figs. 1C, 2C). Among the subsequent branches, a variable configuration of branches was observed: the right circumflex branch (*ramus circumflexus dexter*), the proximal intermediate and distal right ventricle branches, and the marginal branch of the right ventricle (*ventriculus dexter*). Ventricular branches were also characterized by high individual variability, mainly related to the presence or absence of their branches (Figs. 1A-D, 2A-E).

Due to the high variability in the course of RCA regarding, among the others, the presence or absence (Fig. 2A) of the subsinuosal interventricular branch (*ramus interventricularis subsinuosus*) and branches of the sinoatrial node (*nodus sinoatrialis*), as the determinant of coronary arteries dominance were taken the length of individual branches and the area of their vascularization. If there is a posterior interventricular artery (*a. interventricularis subsinuosus*), it is always a branch of the LCA (Fig. 2F).

These observations allowed us to distinguish the two most common variants of the RCA course. Type 1 (n = 11) consists of cases where, from the trunk of the RCA running in the coronary sulcus (*sulcus coronarius*), the conal branch of the pulmonary trunk and the sinoatrial nodal branch that supplies the upper wall of the right atrium and the area of the sinoatrial node branched off in turn (Fig. 2B). The right circumflex branch, continued as an extension of the RCA under the right auricle towards the coronary sinus (*sinus coronarius*) (Fig. 2B). Without reaching the subsinuosal interventricular sulcus, the right surrounding branch of RCA gave off small branches to the wall of the coronary sinus, and in the further course a variable number of right ventricular secondary branches. These vascularized the proximal third of the right ventricular wall. In this type, the marginal branch of the right ventricle and the typical subsinuosal interventricular branch were not seen (Fig. 2A).

In type 2 (n = 15) the biggest branch was the marginal branch of the right ventricle (Figs. 1D, 2C). This vessel followed the sharp edge of the heart and supplied the proximal part of the right ventricle wall. In a few cases (n = 3) this constituted a continuation of the distal RCA segment. In this case, the right circumflex branch was underdeveloped. The branches of the right coronary artery vascularized about three quarters of the proximal part of the right ventricular wall. The remaining distal part of the right ventricular wall was supplied by the terminal branches of an extremely highly developed septal branch extending from the left coronary artery trunk (Figs. 1C, D, 2D, F). In its course through the interventricular septum (*septum interventriculare*), this vessel, in the middle of its length, radiated branches supplying the interventricular septum. The distal part of the LCA septal branch emerged on the surface of the right ventricular wall near the apex of the heart, where it ended in numerous small ramifications within the right ventricular wall.

In a few cases (n = 3), intermediate variants were observed where the marginal and the surrounding branches were similarly developed (Figs. 1A, 2D). One case involved a fetal heart with an unusually branched RCA. In this case, the short trunk was divided into four main branches: atrial, marginal, septal, and right circumflex. This last showed a very distinct nodal branch (Fig. 2E).

The left coronary artery departed in all cases (n = 30) from the left aortic sinus at the level of the free edge of the semilunar valve and ran towards the coronary sulcus (Figs. 1A, B, C, D, F, 2A, D, F). The left coronary artery was the dominant vessel with respect to the RCA, both in terms of diameter and area of vascularization (Fig. 2A, F). The average diameter of the LCA trunk measured at the wall of the aortic bulb was 0.74 mm (± 0.16). The LCA in its course divided into interventricular paraconal branch, circumflex, septal and ventricular branches. A wide range of variability in this vessel was also observed regarding both the length of the trunk and the number of branches. There were no sex differentiations of the examined structures.

## Discussion

The coronary vascularization of vertebrate hearts has been the subject of extensive research involving many groups of animals, including exotic species and specific breeds within a given species (Atalar et al. 2003; Hagensen et al. 2008; Yuan et al. 2009). Most descriptions have naturally focused on human coronary artery pathology (Kang et al. 2006; Gleeson et al. 2009; Sanyal et al. 2012). There are reports in the literature of the presence of the single coronary artery in a hamster and dog which is a result of malformations (Durán et al. 2006, 2007a, b, 2009; Owens et al. 2021). Apart from this, some publications have compared the normal vascularization of the heart of animals, such as pigs, with that of humans (Barszcz et al. 2013). In the hearts of mammals, the course of the coronary arteries may be predominantly intramuscular (rat, guinea pig, hamster). Predominantly epicardial course, with occasional intramural parts, is found in human, sheep, dog, and cat. Exclusively epicardial course has been observed for horse, cow, and pig (Bil 2016).

The occurrence of a single coronary artery has been described in human. This seems to be a birth defect and is often associated with other congenital heart defects (Hauser 2005).

Our attention was drawn to the results presented by Özdemir et al. (2008), which clearly indicated that the chinchilla’s heart is only vascularized by the left coronary artery. According to these authors, the right coronary artery was absent from nine out of ten cases they examined. In one case, there was a vessel that was too underdeveloped to be called a normal right coronary artery. The results of Özdemir et al. (2008) clearly suggest that this is not an anomaly, but a normal vascular pattern in chinchillas. Their study was the first to report the unilateral absence of a coronary artery from the normal anatomical model of a higher vertebrate.

Our observations, however, do not confirm the results of Özdemir et al. (2008). Although the LCA was found to dominate, normal RCA was unequivocally found in all 30 examinations, in which the coronary vessels were filled with colored latex and visualized using contrast micro-CT.

Generally, it is possible for either the LCA or the RCA to be dominant, in the sense of giving off the SA nodal artery. Most of the time the right coronary artery is dominant in man (Vikse et al. 2016). The left coronary artery is dominant in the chinchilla, while the sinoatrial nodal artery (SA) is a branch of the right coronary artery. The separation of the septal artery from the left coronary trunk additionally establishes the dominance of the left coronary artery (Lopez-Garcia et al. 2016). Dominance of the LCA has been noted in dogs and rabbits, while dominance of the RCA has been described in goats, pigs, and 80% of humans (Scansen 2017).

The RCA has also been found to dominate in the capybara (Magariños et al. 2018), a species related to the chinchilla. However, the RCA was in this case much less developed and less branched than its left counterpart. It ran between the pulmonary trunk and the right atrium, and then along the coronal sulcus, but it did not reach the subsinuosal sulcus.

The porcupine (Atalar et al. 2003), like the chinchilla, exemplifies the left-coronary type (left coronary artery dominance). This type has also been found in other domestic and wild animal species (Mia et al. 1978).

Nickel et al. (1981) report that in pigs and horses, the interventricular septum was vascularized by both coronary arteries. In carnivores and ruminants, the interventricular septum was mainly supplied by the left coronary artery. The study of Icardo and Colvee (2001) indicates that, in rats, the ventricular septum is vascularized by the right coronary artery. In contrast, in the porcupine, it was observed that the interventricular septum was vascularized by arteries originating from the left coronary artery (Atalar et al. 2003).

In chinchillas, the septal branch was a strong (big) branch of the LCA vascularizing the interventricular septum, and sometimes also the distal part of the right ventricular wall. The existence of one or two septal arteries is the most consistent feature of the coronary artery system in rodents (Durán et al. 1992).

## Conclusion

The coronary arteries of chinchilla are generally not different from that described in most vertebrates. Our results definitively contradict the observations of Özdemir et al. (2008) which state that: “the heart of chinchilla was supplied by the left coronary artery only” and that “the right coronary artery did not exist, suggesting that this is apparently not an anomaly but a normal pattern for chinchillas”. Therefore, according to our observations, the variability in the course of the coronary vessels in the chinchilla heart is therefore a contribution to further investigations.

## Data Availability

The datasets generated during and analyzed during the current study are available from the corresponding author on reasonable request.
